# eHealth-Enhanced Peer Navigation for Substance Use Treatment and HIV Prevention Service Linkage for Young Adults Surveilled by the Criminal Legal System: Protocol for a Pilot Randomized Trial Study

**DOI:** 10.2196/54815

**Published:** 2024-03-26

**Authors:** Stephanie L Creasy, Sheridan Sweet, Janet J Myers, Martha Shumway, Marina Tolou-Shams, Nicole McCaffrey, Emily F Dauria

**Affiliations:** 1 Department of Behavioral and Community Health Sciences School of Public Health University of Pittsburgh Pittsburgh, PA United States; 2 Department of Infectious Diseases and Microbiology School of Public Health University of Pittsburgh Pittsburgh, PA United States; 3 Division of Prevention Science Department of Medicine University of California San Francisco, CA United States; 4 Department of Psychiatry and Behavioral Sciences School of Medicine University of California San Francisco, CA United States; 5 Department of Psychiatry Zuckerberg San Francisco General Hospital and Trauma Center University of California San Francisco, CA United States

**Keywords:** substance use, HIV prevention, carceral system, intervention development, young adult

## Abstract

**Background:**

In the United States, the proportion of criminal legal–involved (CLI) adults with a substance use disorder reaches 72%, and ~150,000 persons with HIV pass through a carceral setting annually, which represents 16% of the HIV-infected population nationally. Despite the high need for substance use treatment and HIV prevention services, few carceral settings successfully link CLI individuals to treatment upon release. Young adults represent 41.9% of the adults incarcerated in the United States and have the highest HIV incidence rates nationally. Peer patient navigation has successfully increased community-based care linkage for people living with HIV leaving jail; yet, peer-led navigation for HIV prevention among HIV-negative CLI populations is undeveloped and untested. eHealth approaches to substance use and HIV prevention services hold promise because they improve access to effective intervention services, particularly for younger people.

**Objective:**

This paper describes a protocol for a pilot randomized controlled trial that aims to improve linkage to substance use treatment and HIV prevention services using peer navigation and a codeveloped eHealth technology adjunct.

**Methods:**

The three aims of this study are to (1) adapt an existing evidence-based navigator model and incorporate codeveloped eHealth technology to refer and link young adults (18 to 29 years) surveilled by the criminal legal system to substance use and pre-exposure prophylaxis (PrEP) services; (2) refine and test the intervention with criminal legal–involved young adults (CLI-YAs); and (3) assess the feasibility, acceptability, and impact of the intervention. Data to inform the intervention will be collected via system partner interviews (n=4) and focus groups with CLI-YAs (n=24). Next, an open trial (n=10) will be conducted. The intervention will be refined via interviews with participants and facilitators, and a randomized pilot trial (n=75) will be conducted to assess the feasibility, acceptability, and preliminary impact of the eHealth-enhanced navigation on substance use and PrEP services linkage. Exit interviews conducted with a subsample of intervention participants (n=10), the navigator (n=1), and system partners (n=4) will assess intervention acceptability and suggestions for improvement. A community of practice, a group of system partners with an interest in working toward solutions to common problems, will inform each phase of the study.

**Results:**

The project is currently ongoing. The project was funded in September 2022. Internal review board approval was received on March 21, 2022. The first results from early study aims are expected to be published in 2025.

**Conclusions:**

This study provides an opportunity to reduce HIV acquisition and improve access to substance use treatment in a systemically marginalized group: young CLI-YAs. The results will contribute to the development and testing of a future multilevel randomized controlled trial.

**International Registered Report Identifier (IRRID):**

DERR1-10.2196/54815

## Introduction

In the United States, annually, individuals involved in the criminal legal (CL) system are at an increased risk of substance use disorders (SUDs) and HIV acquisition [1-4]. HIV rates among CL-involved (CLI) adults range from 3 to 15 times the rate among individuals not involved in the CL system [2]. Individuals involved in the CL system have high rates of lifetime and recent substance use: between 81% and 84% report lifetime substance use and between 63% and 83% test positive for substance use at the time of their arrest [3]. The proportion of CLI individuals with an SUD reaches 72% [3]. Despite the high need for treatment services, few correctional settings link CLI individuals to community-based substance use treatment upon release.

Young adults (YAs; ages 18-34 years) represent 41.9% of the adults impacted by the carceral system [5] and have the highest HIV incidence rates nationally; YAs aged 20 to 24 years and 25 to 29 years have the highest rates of HIV infections (27.9 and 32.6 per 100,000, respectively) [6]. YAs also disproportionately experience SUD, with 25.6% of those aged 18 to 25 years estimated to meet the Diagnostic and Statistical Manual of Mental Disorders criteria for SUD in the last year (as of 2021) compared to 16.1% of those over the age of 26 years [6]. Factors that increase the likelihood of substance misuse and SUD among YAs include an imbalance of brain maturity through the second decade of life as well as psychosocial factors like increased peer influence and substance use as part of identity exploration [7]. Notably, the criminalization of substance use can also shape vulnerability to HIV as periods of detention can disrupt health care access, employment, caregiving, and social networks [8].

Significant racial disparities exist among new HIV acquisitions for YAs; African American populations accounted for 45.7% of all new HIV infections among YAs, and Hispanic populations accounted for 28.8% [9]. Given the disproportionate number of racial and ethnic minoritized CLI-YAs [10], prioritizing interventions that reduce racial and ethnic disparities in HIV and SUD treatment engagement for CLI-YAs is essential [11,12]. Multiple negative outcomes are known to be associated with involvement with the carceral system itself, including increased prevalence of SUD and infectious diseases like hepatitis C and HIV, increased indicators of poor mental health, and high prevalence of traumatic experiences (physical, sexual, and emotional) that linked directly to carceral policies (eg, solitary confinement) [13,14]. In light of the fact that ~5 million adults pass through CL settings annually and one-third are YAs [10], it is critical to link CLI-YAs who misuse substances and are at risk for HIV with innovative prevention strategies (ie, pre-exposure prophylaxis [PrEP]) and substance use treatment.

PrEP, in the form of a fixed-dose combination of 2 antiretroviral drugs, has emerged as a powerful HIV prevention tool [15]. In addition to 2 oral PrEP medications available (ie, Truvada and Descovy), the US Food and Drug Administration recently approved long-acting injectable PrEP (ie, Apretude) as another PrEP administration tool [15]. Significant disparities exist both between PrEP eligibility and uptake. For example, there are significant racial disparities with low PrEP uptake for Black individuals compared to people with other racial identities [16]. Hispanic and Latinx populations face similar disparities in PrEP use; people assigned female at birth, transgender populations, and bisexual and heterosexual populations also experience lower odds of PrEP use compared to their counterparts despite their not insignificant HIV incidence rates [16]. A variety of factors impede efforts to increase PrEP and substance use treatment access among CLI populations including discrimination based on one’s racial or ethnic identity, misinformation about HIV transmission and treatment, stigma related to an individual’s intersecting identities (eg, CLI status, substance use, race, and ethnicity), distrust of the CL and medical systems, psychiatric symptoms, inability to pay for transportation, lack of familial and social support, and inconsistent access to health care [17-20]. Although PrEP has been found to be efficacious, its maximal impact depends on uptake among those at high risk, including CLI-YAs.

Peer navigation holds strong potential to address multifactorial and complex barriers to PrEP and substance use treatment linkage and uptake for CLI-YAs. Navigation uses a one-on-one relationship to promote the timely movement of an individual through a health care continuum by eliminating barriers [18]. One such intervention, The Navigator Project (NAV), found that navigation-enhanced case management supported linkage to care (<30 days) and consistent retention in care (<1 year) for persons with HIV leaving jail as compared to persons with HIV living in the community and not exposed to jail detention. Using a harm reduction framework, peer navigators use motivational interviewing and prevention care management to navigate reentry back into the community, including referrals for mental health treatment, substance use treatment and harm reduction support, housing, employment, legal aid, and social services [20]. While peer navigation has been successful in increasing connections to care for persons with HIV leaving jail settings [20,21], peer-led navigation for HIV prevention among HIV-negative CLI populations is nascent [22].

In tandem with patient navigation, eHealth has the potential to improve health care engagement for CLI-YAs [23,24]. eHealth-enhanced peer navigation for HIV-infected individuals released from jail has been shown to be effective in maintaining virologic suppression [21]. The private nature of eHealth makes it an appealing modality to deliver components of a navigation intervention and link individuals with stigmatized services (eg, HIV prevention and SUD treatment) [25-27]. Of YAs aged 18-29 years, nearly 100% own a mobile phone and 92% possess a smartphone, making eHealth health intervention approaches feasible [23]. eHealth interventions can improve attendance at health care appointments [28,29], medication adherence [30,31], and behavior changes [32], and can provide support in real time [33]. To our knowledge, however, eHealth-enhanced navigation is untested for integrated substance use treatment and HIV prevention service linkage for CLI-YAs who have returned to the community after incarceration or who are subject to community surveillance. This study aims to address this gap in the literature by examining the feasibility, acceptability, and impact of an eHealth-enhanced peer navigator-led SUD and HIV prevention referral and linkage intervention for CLI-YAs supervised in the community. This paper is intended to provide a thorough summary of this study protocol.

The objectives of this study are to (1) adapt an existing evidence-based navigator mode (NAV) and incorporate codeveloped eHealth technology to refer and link CLI-YAs (ages 18-29 years) to substance use treatment and HIV prevention (PrEP) services; (2) refine the adapted eHealth-enhanced navigator-led substance use treatment and HIV prevention (PrEP) linkage intervention for CLI-YAs and test for fidelity, appropriateness, and satisfaction; and (3) assess the feasibility, acceptability, and impact of the adapted eHealth-enhanced navigator program to refer and link CLI-YAs to substance use treatment and HIV prevention (PrEP) services.

## Methods

### Study Setting

Surveillance data from Allegheny County, Pennsylvania, the study location, indicate that YAs (aged 20-29 years) account for 43% of all new HIV infections [34]. Notably, most adults newly diagnosed with HIV in Allegheny County are from racial and ethnic minority groups (65.8% in 2020) [34], making research in this geographic area generalizable to other high HIV incidence US locales. As of 2020, 30% of individuals who died from drug overdoses in Allegheny County were involved with adult probation and 19% had been booked in a county jail in the prior year. Additionally, Black residents in Allegheny County were disproportionately represented in overdose deaths, with a rate more than 2 times that of White residents [35].

### eHealth Enhancement

The proposed technology is a non-native web-based app that will facilitate resource linkage and communication between the navigator and participants. Expected functions of the eHealth technology include appointment scheduling and reminders, the provision of tailored resources (eg, substance use treatment information), motivational messages, and linkage to support persons. The partner for developing this technology is Chorus Innovations, Inc, which uses a participatory informatics approach that combines best practices from community-based participatory research (eg, equity and power sharing) [36] and user-centered design (eg, active user participation in design) to build technology products [37]. These practices ensure that the user participates in the design process [38], and there is a collaborative and equitable partnership in the research and design process, the process is iterative, and knowledge and action are mutually beneficial to all partners.

### Implementation Research Logic Model

**Figure 1:**
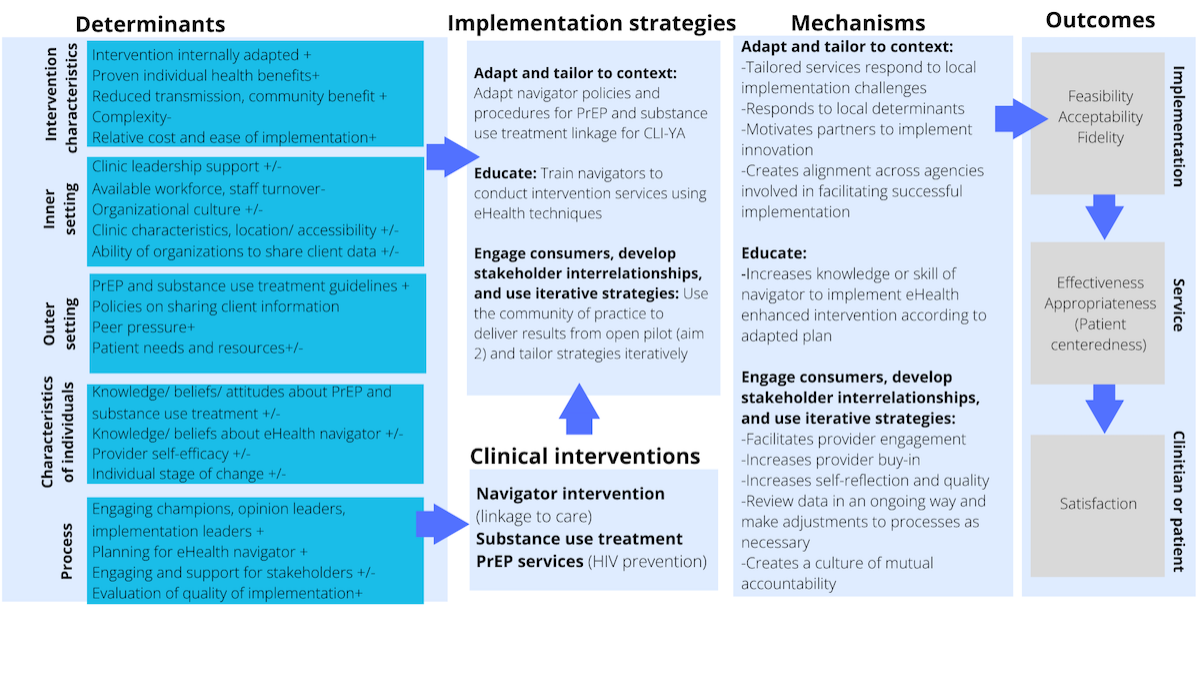
Intervention logic model for a 90-day randomized controlled trial designed to link young adults impacted by the criminal legal system to substance use and HIV prevention services in Allegheny County, PA, based on the CFIR (Consolidated Framework for Implementation Research), Expert Recommendations for Implementing Change (ERIC), and the Proctor Framework. +/- in the determinants column indicates directionality of impact. CLI-YA: criminal legal–involved young adult; PrEP: pre-exposure prophylaxis.

This study uses the CFIR (Consolidated Framework for Implementation Research) to identify key determinants [39], the “Expert Recommendations for Implementing Change” [40,41] to describe implementation strategies, and the Proctor Framework [42] to define implementation, service, and participant outcomes (Figure 1). The CFIR is organized into 5 domains based on context (intervention, outer setting, inner setting, individual, and process). We will use three implementation strategies from Expert Recommendations for Implementing Change [41] to (1) adapt and tailor to context for the eHealth enhancement protocol; (2) education to train navigators; and (3) engage consumers, develop stakeholder interrelationships, and use evaluative and iterative strategies achieved via the community of practice (CoP). This study focuses on feasibility, acceptability, fidelity (implementation outcomes), effectiveness, appropriateness (service outcomes), and satisfaction (clinical outcomes).

### CoP

A CoP, originally conceptualized by anthropologists Lave and Wenger [43], is a community with a shared domain of interest or knowledge that provides support, resources, strategies, and best practices related to the shared domain. Our CoP will meet regularly throughout the intervention and be comprised of individuals from the public health and social service sectors with knowledge of substance use treatment and HIV prevention in CL settings, navigators with previous experience working with CLI-YAs, and YAs with a history of CLI. The CoP will be community experts who will participate in and support all 3 aims of the study including reviewing data collection tools and sampling frames, examining results from each aim, providing guidance on navigation structure and content, codeveloping the adaptation of the Chorus-supported navigation intervention in real time, and guiding research dissemination.

### Aim 1: Development Phase

#### Participants

We present eligibility criteria by separate participant population. CLI-YAs (ages 18-29 years; n=24) will be eligible if they self-report recent CLI (past year); self-identify as HIV negative; endorse behaviors in the past 6 months indicating consideration of PrEP per the Center for Disease Control and Prevention’s risk indices [44]; meet the Tobacco, Alcohol, Prescription medication, and other Substance use Tools’ criteria for identifying the Diagnostic and Statistical Manual of Mental Disorders SUD (ie, ≥2 for tobacco, alcohol, and marijuana, and ≥1 for other substances) [45]; and are conversant in English. Exclusion criteria include being on PrEP, cognitive delays that would interfere with consent or participation, or inability to provide contact information for ≥2 locator individuals. CL system partners (n=2) will be in probation administration or frontline staff (eg, probation officers). Medical or public health system partners (n=2) will be involved in substance use treatment delivery and PrEP participant care in a setting where CLI participants are referred and will include individuals who have had experience navigating CLI to health services.

#### Recruitment

CLI-YAs will be recruited for participation in focus groups. To recruit focus groups, study staff are partnering with community-based carceral settings and community-based organizations serving CLI-YAs. Study staff will approach YAs for participation when they (1) come to meetings with their probation officers or (2) access services at community-based agencies serving CLI-YAs.

System partners will be recruited for individual interviews. Recruitment will be conducted using purposive sampling of community partners via email and phone outreach. We will identify initial contacts who work in CL and public health or health care spaces relevant to the study and use snowball sampling to include additional unknown system partners.

#### Data Collection

##### Overview

Focus groups and interviews will be held at times and locations convenient for participants (eg, for CLI-YAs, around probation visits, work schedules, or on weekends; for system partners, during lunch, or after work). Focus groups will be held via Zoom (Zoom Technologies Inc) or in a private room depending on participant preference and availability, while individual interviews will be conducted by phone; both will be audio-recorded. A semistructured focus group and individual interview format will allow participants to respond freely and in an open-ended way [46].

##### Focus Groups

The research team will conduct 3 separate (75-90 minutes) focus groups with CLI-YAs (n=24). Focus groups will be informed by 2 domains of the CFIR relevant to CLI-YAs: characteristics of the intervention and individuals involved in the intervention. Sample content areas will assess the (1) knowledge about navigation as an effective approach to substance use treatment and HIV prevention service linkage, (2) perceptions of relevance of *NAV* content and gap areas, (3) strategies to support the likelihood of engaging with a navigator throughout the intervention period, and (4) attitudes toward and capabilities of using eHealth intervention enhancements of the CLI-YAs.

##### Interviews

As with the focus groups, individual interviews will be guided by several domains from the CFIR (eg, inner setting, intervention characteristics, and implementation process). Sample interview topics include (1) how a navigation intervention could be adopted, delivered, and sustained within the probation system; (2) perceptions of what YAs do or do not like to hear from them and what they might or might not like to have the navigator do on the topics of substance use, HIV risk, PrEP eligibility, and linkage; (3) identify intervention practices that might facilitate information sharing within and across probation and medical system while respecting participant privacy and confidentiality; and (4) areas of training relevant for navigators. At the end of the focus groups and individual interviews, study staff will administer a brief questionnaire to assess the sociodemographic characteristics of the sample.

#### Data Management and Analysis

We will conduct a content analysis, guided by CFIR domains, of notes collected as part of the CoP meetings occurring during this aim, focus groups, and individual interviews. Executive summaries, which provide data quickly, and evidence of whether theme redundancy is achieved, will be written within 48 hours of data collection. Digital recordings of all qualitative data will be transcribed verbatim. Following transcription and the completion of the executive summaries, data will be analyzed according to Inductive Thematic Analysis [47]. Inductive Thematic Analysis is often used to find solutions to real-world problems and provide program recommendations [48]. An initial codebook will be developed based on the focus group and individual interview guides and transcripts. To improve reliability and ensure adequate intercoder agreement, members of the research team will compare coding patterns, and the codebook will be refined until consensus is reached. Study team staff will generate memos to highlight connections between codes and subcodes. The team will meet weekly to discuss data as they are being collected and to come to a consensus on themes. To identify unique developmental, gender, and cultural perspectives, we will also examine data by race, ethnicity, sexual orientation, gender identity, and age or development (18-25 vs 26-29 years). NVivo (QSR International) will be used to facilitate analyses.

#### Outcomes

The primary outcome of aim 1’s formative work is the adaptation of the existing navigator model (NAV) and the codevelopment of the proposed technology for eHealth enhancement. Both will be informed by the CoP and findings from focus groups with CLI-YAs and individual interviews with system partners. Both adaptation of the existing model and navigator training will be completed prior to the open pilot in aim 2.

### Aim 2: Open Pilot Phase

#### Participants

To refine and test the adapted eHealth-enhanced intervention for CLI-YA, we will pilot the intervention with CLI-YAs (n=10). Final eligibility criteria for aims 2 and 3 will be determined from insights gained through the implementation and data collection of aim 1.

#### Recruitment

To recruit participants, study staff will be stationed in probation offices and community-based organizations working with individuals involved in the carceral system and will approach individuals to ask if they are interested in participating in the study screening. After consent, the study staff will refer participants to the navigator who will make contact within 24 hours.

#### Pilot Intervention Navigation Protocol

The navigation process will be informed by evidence-based practices from NAV, including harm reduction, peer navigation, motivational interviewing, and prevention care management to navigate reentry back into the community (eg, referrals for mental health treatment, substance use treatment, and social services) [20]. Navigation processes will be informed by 2 cascade frameworks: the *PrEP Continuum of Care* model [49] and the *Juvenile* *Justice* *Behavioral* *Health Services* *Cascade* [50]. The PrEP continuum emphasizes identifying individuals at risk for HIV acquisition, increasing awareness of and willingness to take PrEP, ensuring linkage and access to PrEP, and adherence to PrEP medications. The *Juvenile Justice Behavioral Health Services Cascade* [50] was developed to move CLI youth from substance use screening and identification in CL settings to community-based treatment initiation and engagement; this cascade will be adapted to reflect processes specific to YAs.

Navigators will act as role models who can steer participants through the upstream steps of these cascades from screening to linkage with an exploratory look at engagement or initiation, adherence (PrEP specific), and continuing care (substance use treatment) or persistence (PrEP specific). We will test a 30-day intervention period (Textbox 1 for sample navigator visits, adapted from *NAV*). The first session (~75 minutes) will occur within 5 days of referral. In line with the *NAV* protocol, the navigator will initiate contact with the participant ≥3 times per week after the first session (the number of contacts may be higher, depending on the participant’s needs and desires) [20]. Additional contacts could be made throughout the 30-day period in-person or with support via Chorus (eg, SMS text messages). Visits can occur via Zoom, telephonically, or in person based on the participant’s preferences. To maximize program retention, each navigation session will begin by updating the participant’s contact information. If detention interrupts intervention delivery, we will “reset,” where the participant left off to complete 30 days of exposure. Intervention “graduation” will be acknowledged and included in each participant’s action plan (eg, certificate of completion).

**Sample navigator session content for a 90-day randomized controlled trial designed to link young adults impacted by the criminal legal system to substance use and HIV prevention services in Allegheny County, PA.**

**Session 1: Program orientation, relationship building, and needs assessment (~75 minutes)**

- The navigator will build rapport by introducing themselves, their role, and the program. The navigator will identify significant contacts, and complete study documentation (eg, release of information forms). Finally, the navigator will conduct a comprehensive needs assessment of sexual health and substance use to develop an individual risk reduction plan. Relevant community resources will be presented to the participant.
- eHealth approaches identified in aim 1 (eg, SMS text messages, appointment reminders, motivational messages, and telehealth sessions) will be incorporated into session content.

**Session 2: Substance use education and treatment cascade (~60 minutes)**

- Provide psychosocial education on substance use (tailored to the client’s behaviors and needs identified in session 1), provide participants with information on how patterns of substance use can affect their health, social, and legal outcomes and the Juvenile Justice Behavioral Health Services Cascade. The navigator will revisit the risk reduction plan (session l) and tailor it to any changes in the participant's life. Participants will be referred to local substance use treatment.
- eHealth approaches identified in aim 1 (eg, SMS text messages, appointment reminders, motivational messages, and telehealth sessions) will be incorporated into session content.

**Session 3: HIV and Pre-exposure Prophylaxis knowledge and care cascade (~60 minutes)**

- Provide psychosocial education on sexual health (tailored to the client’s behaviors and needs identified in session 1) and can shape an individual’s risk of sexually transmitted infections and HIV. Provide basic HIV (eg, transmission) and pre-exposure prophylaxis information, and outline the pre-exposure prophylaxis Care Continuum. The navigator will revisit the risk reduction plan (session 1) and tailor to any changes in the participant’s life. Participants will be referred to local pre-exposure prophylaxis–related services.
- eHealth approaches identified in aim 1 (eg, SMS text messages, appointment reminders, motivational messages, and telehealth sessions) will be incorporated into session content.

**Session 4. Substance use and Pre-exposure Prophylaxis care appointment scheduling and goal setting (~60 minutes)**

- Provide follow-up information on where and how to access substance use and pre-exposure prophylaxis–related services. Develop a plan for substance use and pre-exposure prophylaxis linkage and maintenance to include planning for transportation to health care services, keeping appointment dates, obtaining medication (pre-exposure prophylaxis), formulating a medication schedule, and identifying motivation for appointment attendance and pre-exposure prophylaxis adherence.
- eHealth approaches identified in aim 1 (eg, SMS text messages, appointment reminders, motivational messages, and telehealth sessions) will be incorporated into session content.

**Follow-up contacts (~30 minutes)**

- Maintain regular contact between the navigator and the participant, provide guidance and support for goal setting, and ongoing emotional and psychosocial support.
- eHealth approaches identified in aim 1 (eg, SMS text messages and telehealth) will be incorporated to support follow-up contacts.

#### Navigator Training

The Navigator will receive a minimum of 7 days of training focused on relational service provisions (ie, emotional and psychosocial support), logistical service provisions (ie, assistance to overcome task-oriented barriers like scheduling and finding transportation to appointments), technology training, and population-specific training (eg, carceral system processes and harms perpetuated by the carceral system) [51]. Aim 1 system partner interviews will inform additional areas of training relevant to implementing a peer-led model, as well as preferred navigator characteristics (ie, peer navigators will share an age range and at least 1 intersecting identity or experience such as CLI, PrEP use, or substance use treatment with participants).

#### Intervention Fidelity

During the open pilot, navigators will audio record and complete a checklist documenting the delivery of session material [52]. Navigators will engage in weekly supervision with the principal investigator, and the principal investigator will periodically audit records and conduct field visits to observe sessions (required activities will be rated for completeness using a 4-point scale ranging from 0 “not at all” to 3 “completely”).

#### Exit Interviews

The research team will collect qualitative feedback on the open pilot regarding intervention content, structure, and delivery (type of contact, ie, in-person or over the phone, eHealth [Chorus] enhancement acceptability, and a number of contacts) via individual interviews with CLI-YAs. We will also obtain feedback from system partners regarding intervention recruitment, referral, delivery, and implementation. The navigator will be actively involved in the intervention pilot testing as well as in making further intervention manual revisions according to their lived experience of navigation delivery. Exit interview data will be analyzed using rapid qualitative analysis methods to reduce the time completing analysis to ensure timely results for use in aim 3; rapid analysis has been shown to have considerable overlapping findings with traditional qualitative analytic approaches [53,54].

#### Open Pilot Outcomes

In addition to completing a 7-day minimum navigator training, the open pilot’s primary outcome is qualitative feedback to assess appropriateness and satisfaction. The results will be used to refine the intervention for pilot testing in aim 3.

### Aim 3: Pilot Randomized Controlled Trial Phase

#### Participants

To assess the feasibility, acceptability, and impact of the adapted eHealth-enhanced intervention for CLI-YAs, we will test the intervention in a randomized pilot trial with CLI-YAs (n=75).

#### Recruitment and Randomization

**Figure 2:**
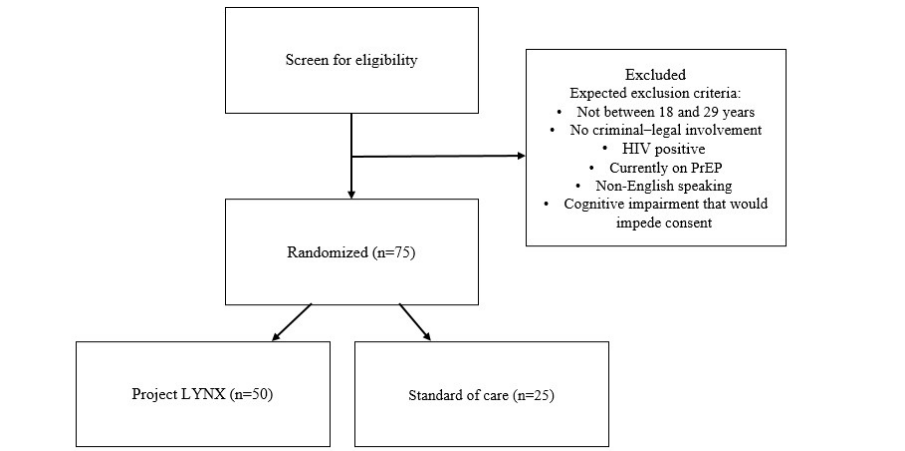
Randomization protocol for aim 3, a 90-day randomized controlled trial designed to link young adults impacted by the criminal legal system to substance use and HIV prevention services in Allegheny County, PA. PrEP: pre-exposure prophylaxis.

**Figure 3:**
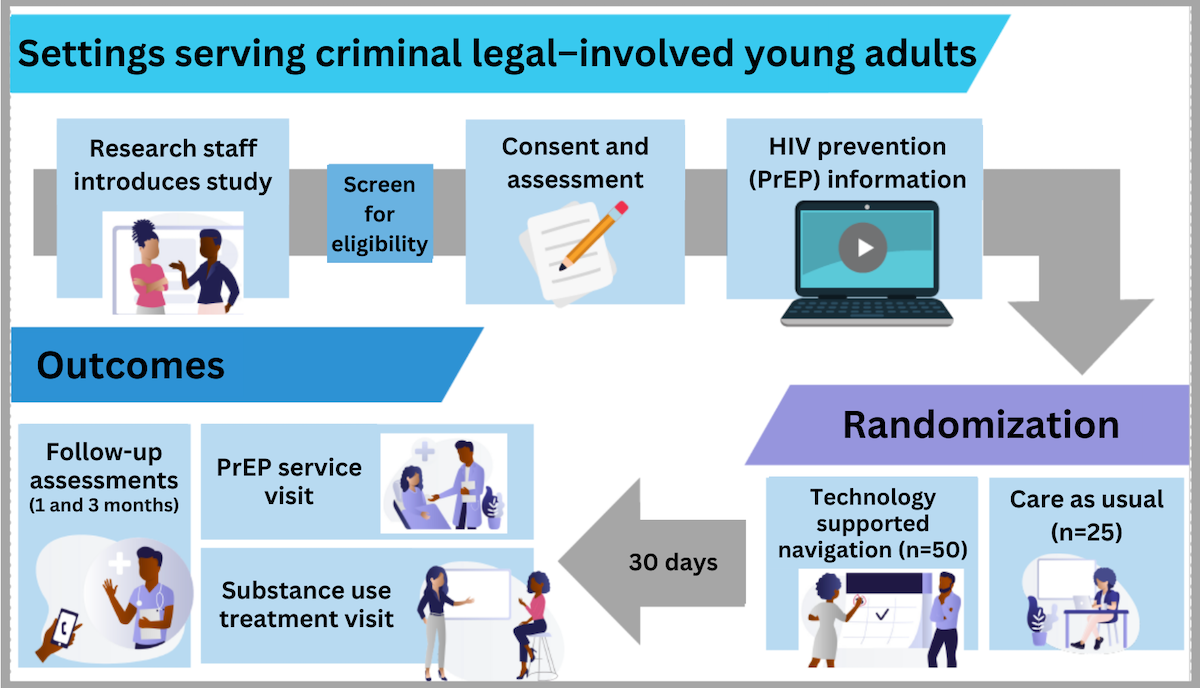
Recruitment, randomization, and intervention practices for aim 3, a 90-day randomized controlled trial designed to link young adults impacted by the criminal legal system to substance use and HIV prevention services in Allegheny County, PA. PrEP: pre-exposure prophylaxis.

Recruitment procedures will be informed by aim 2 results. Participants will be randomized either to the intervention group (n=50) or the standard of care (n=25) using a 2:1 randomization allocation scheme (Figure 2). Figure 3 provides a schematic of our study processes. Each participant will complete a baseline assessment immediately following the consent process. At the end of the baseline assessment, participants will be randomized to the intervention or control condition using a computerized algorithm programmed into REDCap (Research Electronic Data Capture; Vanderbilt University). Randomization will be blocked to avoid “runs” of assignment to the same.

#### Standard of Care

The standard of care is a referral to substance use treatment and HIV prevention services from an individual’s case worker.

#### Data Collection

The baseline assessment will occur immediately following participant consent, prior to randomization, and follow-up assessments will occur at 1 month and 3 months (Figure 3). Data will be collected via face-to-face one-on-one interviews in private locations to ensure confidentiality. If face-to-face interviews are not possible, a participant will be given the option of completing the assessment via a secure web-based survey link. Regardless of the method of interview administration, REDCap will be used to collect data (ie, in person on tablets or via a secure REDCap survey sent via email). If completing the assessment via a secure link, specific instructions will be provided to the participant to inform them about how to keep their data private.

#### Pilot Trial Outcomes

Primary outcomes are linkage to substance use treatment and PrEP services. Because PrEP and substance use treatment health service linkage are a process and not a 1-time event, we will also explore prelinkage (eg, navigator satisfaction) and downstream outcomes (eg, PrEP initiation, adherence, and substance use treatment continuing of care) in an exploratory fashion [55]. The navigator will track these outcomes, but the primary outcomes of (1) PrEP linkage will be attendance at a PrEP services appointment, verified by medical records and (2) substance use treatment linkage will be attendance at a substance use treatment appointment, verified by the navigator and medical provider collateral (if the participant agrees). Other exploratory outcomes include time to health service linkage (substance use and PrEP) and data on HIV testing at the PrEP linkage appointment (uptake, result). Participants will be asked whether and for how long they have experienced hospitalization, residential treatment, and detention since the intervention to account for any system barriers to linkage. We will also assess preferred methods for communicating with the navigator as well as participant satisfaction with the intervention and navigator services. Table 1 provides additional detail on outcomes and correlates.

**Table 1:** Description and schedule of outcomes from a 90-day randomized controlled trial designed to link young adults impacted by the criminal legal system to substance use and HIV prevention services in Allegheny County, PA.

Primary outcomes		Baseline		30 days		90 days
PrEP^a^ linkage: medical record				✓		✓
Substance use treatment linkage: self-report, medical provider collateral if the participant agrees				✓		✓
**Secondary outcomes**						
	Navigator satisfaction: NAVSAT Pt 1		✓			
	Client intervention satisfaction: Study Participant Feedback Questionnaire	✓	✓		✓	
	PrEP care continuum (accepted, initiated, and adhered): navigator report, medical record, and self-report		✓		✓	
	Substance use treatment continuum (accepted, initiated, and adhered): navigator report, medical record, and self-report		✓		✓	
	Linkage to other services: self-report and Navigator report		✓		✓	
**Sociodemographic, psychosocial, and behavioral correlates**						
	Demographic information (age in years, race or ethnicity, legal history, etc)	✓			✓	
	Health care use	✓			✓	
	Medical mistrust: Health Care System Distrust Scale	✓			✓	
	HIV knowledge or attitudes or risk: HIV-related knowledge scale, HIV-related attitudes scale, HIV stigma scale	✓			✓	
	PrEP knowledge or attitudes: willingness, intentions to use, barriers, stigma or conspiracy beliefs	✓			✓	
	Substance use: Alcohol use Disorders Identification Test, Texas Christian University Drug Screen V, Internalized Stigma of Substance Abuse	✓			✓	
	Psychiatric symptoms: Global Appraisal of Individual Needs-Short Screener	✓			✓	

^a^PrEP: pre-exposure prophylaxis.

#### Data Management and Analysis

REDCap will facilitate routine data checking and exportation of data for analysis. Analyses will be conducted using SAS (version 9.4; SAS Institute). Preliminary analyses will include the examination of descriptive statistics, distributions, and internal consistency reliability of scaled measures. Logistic regression will be used to examine the relationship between the condition and the primary outcomes (services linkage, ie, PrEP, substance use treatment), and all other dichotomous outcomes (eg, acceptance of PrEP and substance use treatment referral). Dependent variables in initial analyses will be summary dichotomous indicators based on both month 1 and 3 assessments, reflecting whether the outcome ever occurred. Subsequent analyses will separately examine whether outcomes occurred by month 1 and by month 3. Generalized linear models will be used to examine variously distributed continuous outcomes (navigator contacts, time-to-service linkage, and satisfaction). Exploratory analyses will build on these models to preliminarily examine the impact of potential moderating variables including sociodemographic characteristics, HIV risk behaviors and perceptions, PrEP-related knowledge and attitudes, substance use treatment behaviors, psychiatric symptoms, social support, and substance use on primary and secondary outcomes.

#### Exit Interviews

We will conduct exit interviews with a subsample of intervention arm participants (n=10), the navigator (n=1), and partners from participating systems (n=4). Interviews will assess intervention acceptability and suggestions for improvement. To recruit for qualitative exit interviews, a member of the study team will contact intervention participants to invite them to a phone interview (~30 minutes). To ensure a range of responses, we will sample for (1) participants who connect or did not connect to the navigator and who linked or did not link to PrEP and substance use treatment services and (2) variation in characteristics that may impact the intervention (eg, psychiatric symptoms). We will seek to understand reasons why the intervention was or was not successful for a particular participant (via participant and navigator report). To analyze exit interview data, we will mirror aim 2’s rapid qualitative analysis processes.

### Ethical Considerations

#### Overview

This study was approved by the University of Pittsburgh internal review board (reference 22020053). As the intervention is refined, changes to the protocol will be submitted to the internal review board for further approval. Additional protections for participants are provided via a Certificate of Confidentiality through the National Institute of Health. This certificate allows the researchers to legally refuse to disclose information that may identify participants, even by a court subpoena, in any federal, state, or local civil, criminal, administrative, legislative, or other proceedings. To ensure confidentiality, all data will be deidentified and participants will be assigned a unique participant ID. Participant contact information will be stored separately from any data that have been collected (eg, screening surveys, transcripts, and assessments).

#### Aim 1: Development Phase

##### CLI-YA

To determine eligibility, participants will undergo a confidential screening survey with study personnel. Following screening, participants will be compensated US $10 regardless of eligibility status. Eligible participants will undergo a verbal consent process with study personnel prior to participating in focus groups. The consent process will include (1) a review of the information sheet in detail, (2) an explanation of all study procedures in full detail, and (3) an opportunity for the participant to ask questions and have them answered. Potential participants will be asked to paraphrase the information sheet and will be asked basic questions to determine their understanding of key elements of the informed consent. Participants will be compensated US $40 following focus group participation.

##### System Partners

Prior to participating in interviews, system partners will undergo a verbal consent process that follows the same protocol as the focus group consent process outlined above. Participants will be compensated US $50 following completion of an individual interview.

#### Aim 2: Pilot Trial Phase

Prior to participation in the pilot trial, CLI-YAs will undergo a 2-step consent process. Potential participants will be given an information sheet and provide verbal informed consent for screening. Next, should individuals be interested in participating and eligible based on their HIV risk assessment and SUD screening, they will provide written informed consent to allow participation in the open pilot navigation intervention and exit interview. Participants will be compensated US $40 for participating in exit interviews.

#### Aim 3: Assessment Phase

The consent process for aim 3 follows the same 2-step process as outlined above for aim 2. Participants will be compensated US $40 for the baseline assessment, US $20 for the 1-month assessment, US $40 for the 3-month assessment, and US $30 for participating in exit interviews.

## Results

The project was funded in September 2022. Internal review board approval was received on March 21, 2022. Data collection for aim 1 began on April 19, 2023, and is ongoing. At the time of publication, 5 system stakeholders and 16 CLI-YAs have been enrolled in aim 1 data collection activities. The first results from early study aims are expected to be published in 2025.

## Discussion

### Principal Findings

This study provides an opportunity to reduce HIV acquisition and improve access to substance use treatment in a systemically marginalized group of YAs. This emerging area of research has been rapidly amplified in the context of the ongoing coronavirus pandemic, for which empirical study of effective adjunctive technology tools to promote increased substance use and HIV prevention services access is greatly needed. The results will contribute to the development and testing of a future multilevel randomized controlled trial targeting CLI-YAs, CL system partners, and treatment providers.

### Gaps in Literature and Comparisons With Prior Work

Previous studies leveraging peer navigation as a mechanism to link CLI individuals to HIV care have proved efficacious [19,20]. Patient navigation interventions led by individuals who share key personal characteristics, circumstances, or qualities with their participants (eg, ethnic background, culture, and membership in a subpopulation [formerly engaged in transactional sex or IDU]) or “peers” are ideal because they have demonstrated efficacy in building trust and reducing stigma and discrimination-related barriers to health care engagement, particularly among vulnerable populations [24,56]. The inclusion of eHealth technology in patient navigation is appealing because it provides a discrete platform for individuals to engage with potentially stigmatizing services (eg, substance use treatment and HIV prevention services). It also has the potential to facilitate frequent, meaningful communication between individuals and their peer navigators. Previous studies have found eHealth-enhanced peer navigation effective in maintaining viral suppression among people living with HIV [21]. However, there has been little research using this framework to link CLI-YAs to HIV prevention and substance use treatment services. This study will fill an important gap in the literature and provide insights that will inform future efforts to improve service linkage for CLI-YAs, a vulnerable and structurally precarious population [57].

### Strengths and Limitations

A core tenet of this study is the codevelopment of the eHealth technology and navigation protocol. Including the perspectives of individuals who are a part of the population of focus, in addition to system partners, ensures the intervention will be reflective of the wants and needs of the community it will be serving. The inclusion of system partners and people with lived experience in the CoP is important but comes with challenges due to encounter challenges related to power dynamics between these groups. For example, individuals with lived experience may be hesitant to share their thoughts and opinions when they are in a room of service providers and system partners with whom they might already be familiar. The study team will begin each meeting with an overview of agreements and expectations and moderate the discussion to mitigate issues as they arise; however, we acknowledge the challenge that navigating these relationships might pose.

Additionally, the technology being developed will not be a native app. The benefit of this is that it will be accessible from a mobile device or a computer, which ensures that participants will be able to access the technology whether they have regular access to a mobile device. However, data will not be stored directly on a participant’s personal device, so it will not be accessible without an internet connection. This could be a limiting factor for participants who have a phone but do not have regular internet access.

The inclusion of the eHealth portion of the study has the potential to expand access to linkage services for CLI-YAs. This is especially important because CLI-YAs experience structural and institutional obstacles that can hinder their ability to regularly engage in services [57]. However, there is no funding to distribute phones to participants if they do not have them. This may limit the eHealth functionalities available to participants who do not have phones (eg, SMS text messaging) and may make it difficult for them to engage in the intervention in the same way as participants with their personal devices. We will measure where and how participants are engaging with the technology to better examine the impact of technology access.

### Conclusions

This study provides an opportunity to reduce HIV acquisition and improve access to substance use treatment in a vulnerable, underserved group of young adults (CLI-YAs) by adapting an evidence-based navigation intervention and incorporating eHealth technology. The need to further this line of research has been rapidly amplified in the context of the COVID-19 pandemic, for which empirical study of effective adjunctive technology tools that can promote access to substance use and HIV prevention services is greatly needed. The results will contribute to the development and testing of a future multilevel randomized controlled trial targeting CLI-YAs, CL system partners, and substance use treatment and PrEP providers.

## Appendix

Multimedia Appendix 1 Peer review grant documentation from the National Institute on Drug Abuse.

## Abbreviations

CFIR: Consolidated Framework for Implementation Research
CL: criminal legal
CLI: criminal legal involved
CLI-YA: criminal legal–involved young adult
CoP: community of practice
NAV: The Navigator Project
PrEP: pre-exposure prophylaxis
SUD: substance use disorder
YA: young adult

## Data Availability

The data sets generated in this study are available from the corresponding author upon reasonable request.
