# Supplementary material for: eHealth-Enhanced Peer Navigation for Substance Use Treatment and HIV Prevention Service Linkage for Young Adults Surveilled by the Criminal Legal System: Protocol for a Pilot Randomized Trial Study
Source: JMIR Res Protoc. 2024 Mar 26;13:e54815. doi: 10.2196/54815 (PMC11005443; doi:10.2196/54815)
Supplement: Multimedia Appendix 1 [file resprot_v13i1e54815_app1.pdf]

1R34DA054853-01 Dauria, Emily

RESUME AND SUMMARY OF DISCUSSION: The applicant will test the feasibility and acceptability of an eHealth adapted navigator program to refer and link justice involved young adults (18 to 29 years) with substance use disorder (SUD) to substance use treatment and HIV-prevention services. The premise of this application is strong; SUD is highly associated with involvement in the criminal justice system, and a high risk of being infected with HIV due to the large number of persons with HIV that cycle through the correctional system every year; an intervention that would address SUD among criminal justice involved youth would lower the consumption of drugs, increase PrEP or ART adherence resulting in lower HIV infections and transmission. The application is very significant in its focus on CJI with SUD and promises to have a significant impact on the health of this population. Reviewers were enthusiastic about the many strengths of the application which included a very strong PI and team of collaborators with the expertise in criminal justice population, implementation science, and substance abuse necessary to carry-out this project; the current project builds on extensive preliminary work by this team, particularly a project with the San Francisco Probation and Juvenile justice. The application is guided by numerous implementation science models (CFIR, Proctor) and has assembled a community of practice to support decision making. Nevertheless, the following minor to moderate concerns reduced the high enthusiasm for the application moderately: the procedure regarding the eHealth component are unclear; though the navigation program builds on the team's prior work, the number of sessions/timing are not well justified; it is not clear how the adapted intervention will increase communication and effectiveness; in the data analysis, and it is hard to distinguish between intervention components and strategies. Despite these concerns, the committee is confident that the proposed study will make a significant health impact in the lives of CJI with SUD; consequently, the great majority of reviewers assessed its potential impact as high and the remainder as moderately high.

DESCRIPTION (provided by applicant): There is a strong scientific premise for the study of integrated substance use disorders (SUDs) and HIV- prevention interventions for criminal justice-involved (CJI) populations. SUDs lead to increased rates of justice involvement as well as increased risk for HIV-acquisition. Estimates of the proportion of the CJI-population with a SUD reach 72%, and ~150,000 persons with HIV pass through a correctional facility annually. Although pre- exposure prophylaxis (PrEP), is a proven effective intervention for HIV-prevention, its maximal impact depends on uptake, and adherence among those at high risk. To date, few published studies have examined PrEP uptake among CJI populations. Our ongoing work with CJI women (San Francisco, CA) has identified high rates of risk behaviors (including substance use), low levels of PrEP awareness, and high levels of interest in PrEP once learning of it. Participants reported a strong interest in participating in a navigator-led intervention to screen and link them to PrEP-related services; younger participants (i.e., those aged 18 to 29 years) were eager to incorporate electronic health (eHealth) into navigation services. Navigator models use a one-on-one relationship to promote the timely movement of an individual through a health care continuum by eliminating barriers and have successfully increased healthcare access for CJI HIV-positive adults and individuals with SUDs. eHealth approaches to SUD and HIV prevention also hold promise because they improve access to effective intervention services, particularly for younger people. As a multidisciplinary team with expertise in behavioral health (substance use and HIV prevention) among justice-involved populations, and eHealth supported and navigator- led healthcare access and engagement interventions, we propose to develop and test an eHealth enhanced, navigator-led substance use and HIV-prevention referral and linkage intervention for CJI-young adults. Study aims are to: 1) Adapt an existing evidence-based navigator model (The Navigation Project) to incorporate codeveloped eHealth technology, to refer and link CJI young adults (aged 18 to 29 years) to substance use treatment and HIV-prevention services; 2) Refine and test the adapted, eHealth enhanced, navigator-led substance use treatment and HIV prevention intervention for CJI-young adults for fidelity,

satisfaction, and appropriateness; 3) Assess the feasibility, acceptability, and impact of the adapted eHealth enhanced, navigator program to refer and link CJI-young adults substance use treatment and HIV-prevention services. The proposed study has the potential to: 1) reduce HIV-acquisition and improve access to substance use treatment among a high-risk, underserved group of young adults in the US; 2) test the feasibility, acceptability, fidelity, effectiveness, appropriateness, and satisfaction of implementing an eHealth enhanced, navigator-led substance use and HIV- prevention intervention, and 3) create an intervention suitable for large-scale efficacy testing and translation to other criminal justice settings.

PUBLIC HEALTH RELEVANCE: Young adults involved in the criminal justice system are at an increased risk of substance use disorders and HIV-acquisition. Given the disproportionate number of racial/ethnic minority young adults, criminal justice settings provide an opportunity to reduce racial/ethnic disparities in HIV and substance use treatment engagement by linking these young adults with innovative biomedical HIV prevention strategies (e.g., pre- exposure prophylaxis) and substance use treatment services. We propose to examine the feasibility, acceptability, and impact of an eHealth enhanced, adapted navigator program designed to refer and link justice involved young adults (18 to 29 years) to substance use treatment and HIV-prevention services.

CRITIQUE 1

Significance: 2
Investigator(s): 2
Innovation: 2
Approach: 4
Environment: 1

Overall Impact: This R34 study involves a plan to develop an eHealth + peer navigation program for SUD treatment and PREP uptake among criminal justice involved young adults (CJI_YA). The study builds on team member's experiences working with similar populations. Aim 1 includes formative work towards adapting an existing evidence-based navigator model for CJI-YA and efforts to add an eHealth component. Aim 2 is an open pilot trial to assess fidelity, appropriateness and satisfaction. Aim 3 is a 2 arm pilot trial (N=75; randomized 2:1 to navigation vs. standard care. Strengths include the study team, working across sectors to intervene with a highly at-risk population, including a variety of stakeholders and incorporation of a number of valuable frameworks (adaptation and implementation science) into the study design, and the likelihood of collecting ample data to inform next steps. Score driving weaknesses are generally minor, including that the prospect of starting PrEP and substance use treatment simultaneously could be overwhelming and thus may not work, and the complexity of the study makes the research plan challenging to follow. Overall, however, this study is well poised to result in important data that will drive HIV and substance use care for CJA-SA.

1. Significance:

Strengths

- Builds on literature in this area and team's prior work indicating interest in this type of intervention
- Includes consideration of sex as a biologic variable
- Study is likely to result in a substantial amount of data to inform future work in this area

Weaknesses

- Is there evidence youth are open to starting PrEP and SU treatment simultaneously? Could be overwhelming
- What is the rationale/supporting data for the 5 session/30-day intervention? Might it be overwhelming for youth to start PrEP and SU care simultaneously?

2. Investigator(s):

Strengths

- Strong team brings expertise in criminal justice populations, substance use, HIV prevention via PrEP, eHealth navigation programs, biostats, justice-involved youth, implementation science

Weaknesses

- None noted

3. Innovation:

Strengths

- Combining navigation for PrEP, eHealth, and substance use treatment for CJI-YA with implementation science and a community-informed approach is novel

Weaknesses

- None noted

4. Approach:

Strengths

- ADAPT-ITT/co-development, followed by open pilot and moderately sized pilot trial (N=50)
- Outcomes: fidelity, appropriateness, satisfaction, feasibility, acceptability, and linkage to HIV prevention and SUD treatment are important
- Conceptual framework is solid
- Emphasis on implementation science/stakeholder inclusion is a strength
- Builds on prior work with San Francisco probation dept and existing capacity for virtual service delivery (i.e., technology capability)
- CoP component increases potential sustainability
- Navigator approach with MI and eHealth
- Meeting the needs of CJI-YA by connecting with probation officers/community-based referral agencies is likely to increase uptake and retention

Weaknesses

- Chorus development is unclear—are the CBPR parts innate to the tech or determined by the team?
- Re: focus groups, since there is already an existing navigator model, why aren't the youth providing input directly related to the existing tool and then commenting on how the eHealth component can support that?

- It is difficult to understand the relationship (in time and data acquisition and analysis) between CoP, Focus Groups, and Interviews and how and when the findings will be integrated
- Who will be eligible to be the navigator?

5. Environment:

Strengths

- UCSF has extensive resources, San Francisco's Getting to Zero Initiative, and the San Francisco County Adult Probation Department also bring excellent resources and are current collaborators with Dr. Dauria and Dr. Tolou-shams

Weaknesses

- None noted

Study Timeline:

Strengths

- Complex study but trial seems doable in time proposed and well justified, particularly given a large # of CJI-YA in the area, the relationships with the probation department is well-established, methods are and that follow-up is limited to 3 months for trial participants
- Substantial time for start-up activities, as overall study will be ongoing for 15 months prior to trial
- 1 year for recruitment is reasonable, considering that the probation department had nearly 1,000 persons < age 30 under their care in 2018, and the estimated eligible pool is 508 (this is likely reasonable since trial sample size is N=75)

Weaknesses

- It is not clear if estimates of eligible youth have been modified to account for youth who may be ineligible due to participation in the team's other trials with this population

Protections for Human Subjects:

Acceptable Risks and/or Adequate Protections

- thoughtful approach

Data and Safety Monitoring Plan (Applicable for Clinical Trials Only):

Acceptable

- minimal risk study and appropriate monitoring plan

Inclusion Plans:

- Sex/Gender: Distribution justified scientifically
- Race/Ethnicity: Distribution justified scientifically
- For NIH-Defined Phase III trials, Plans for valid design and analysis:
- Inclusion/Exclusion Based on Age: Distribution justified scientifically

Vertebrate Animals:

Not Applicable (No Vertebrate Animals)

Biohazards:
Not Applicable (No Biohazards)

Applications from Foreign Organizations:
Not Applicable (No Foreign Organizations)

Select Agents:
Not Applicable (No Select Agents)

Resource Sharing Plans:
Acceptable

Authentication of Key Biological and/or Chemical Resources:
Not Applicable (No Relevant Resources)

Budget and Period of Support:
Budget Modifications Recommended (in amount/time)
Recommended budget modifications or possible overlap identified:

- CO-Is are only at 3%; stat @ 2-4%; likely need more time

CRITIQUE 2

Significance: 1
Investigator(s): 1
Innovation: 2
Approach: 2
Environment: 1

Overall Impact: This meticulous R34 proposes aims to develop an eHealth enhanced navigation model linking criminal legal involved young adults in probation to PrEP and substance use disorder care. The first aim utilizes ADAPT-ITT for adaptation of existing navigation models, the second aim tests and refines the model through an open pilot, and the third aim assesses the feasibility, acceptability and impact of the adapted model in a pilot RCT. The PI and team are well positioned to perform this study given established expertise in this area and population and prior collaborations. Conceptual models are well utilized and the adaptation and development process meaningfully engages criminal legal involved young adults, and treatment and probation stakeholders through each stage. Intervention adaptation, navigation training and supervision, trial protocol, measures and data analysis are robustly described. Approach could be strengthened by quantifying whether eHealth components have additive benefit beyond the navigation model alone for PrEP outcomes, and including

intersectional stigma as a measure. Overall this proposal is excellent and has the potential to have strong impact on a population with great vulnerability to HIV and adverse outcomes related to substance use.

1. Significance:

Strengths

- Criminal legal involved youth have both high risk of HIV and SUD, an issue that is further compounded by racial disparities.
- Prior research has demonstrated benefit in navigation models for HIV care and criminal legal populations, as well as eHealth for HIV treatment / adherence.

Weaknesses

- The study as structured does not test the additive benefit of eHealth over navigation alone, neither of which have been tested in the population for PrEP linkage.

2. Investigator(s):

Strengths

- Very strong PI with current R34 funding for PrEP navigation for CJI populations, very well positioned to perform study as presented given experience in HIV, SUD, CJI behavioral science
- Highly expert co-I team with well-defined roles in eHealth, implementation science and quantitative methods
- Most team members with strong collaboration history

Weaknesses

- None

3. Innovation:

Strengths

- Combining eHealth and navigation for referral and linkage to SUD and PrEP care is novel
- Robust participatory approach to intervention adaptation and development is innovative

Weaknesses

- None

4. Approach:

Strengths

- Appropriate use of conceptual frameworks for adaptation (ADAPT-ITT) and implementation (CFIR, Proctor)
- Content of navigation intervention, training, and supervision, with inclusion of motivational interviewing is excellent
- Intervention adaptation process, qualitative data analysis, open pilot and pilot trial methods are thoroughly described and well-conceived

- Robust engagement of criminal legal involved young adults, treatment and probation stakeholders through each stage of adaptation, development, and revision of the intervention
- Sex as a biological variable is addressed

Weaknesses

- Given potential resource requirement of eHealth intervention, quantifying whether it has additive benefit to the navigation model for PrEP outcomes would be desirable. The analytical approach could be strengthened by modifying the pilot to include a burn in period for this component, or considering this approach for the multilevel RCT.
- Given the opportunity to examine racial / ethnic minority youth and reduce disparities as noted in the proposal, consider adding measures to examine intersectional stigma and experience of discrimination in the context of underlying determinants and treatment access (not addressed in the Internalized Stigma of Substance Abuse scale)
- Specific eHealth interventions somewhat undeveloped (such as how to operationalize telehealth with existing clinical providers) compared to proposed navigation model although this will be the focus of COP
- Relationship with SF DPH SUD services seems less robust compared to other clinical / service partners. Navigators may benefit from formalized link with these services as per example of Ward 86 PrEP coordinator.
- Consider including case management / social work stakeholders in COP
- Consider adding crisis management training for navigators
- References don't match up

5. Environment:

Strengths

- Research environment at UCSF is exceptional.
- Strong relationships with Ward 86 and probation per prior projects and LOSs

Weaknesses

- None

Study Timeline:

Strengths

- Timeline is ambitious but appropriate given the organization and above mentioned strengths in this proposal

Weaknesses

- None noted by reviewer

Protections for Human Subjects:

Acceptable Risks and/or Adequate Protections

- Appropriate for high risk population

Data and Safety Monitoring Plan (Applicable for Clinical Trials Only):

Acceptable

Inclusion Plans:

- Sex/Gender: Distribution justified scientifically
- Race/Ethnicity: Distribution justified scientifically
- For NIH-Defined Phase III trials, Plans for valid design and analysis: Not applicable
- Inclusion/Exclusion Based on Age: Distribution justified scientifically

Vertebrate Animals:
Not Applicable (No Vertebrate Animals)

Biohazards:
Not Applicable (No Biohazards)

Applications from Foreign Organizations:
Not Applicable (No Foreign Organizations)

Select Agents:
Not Applicable (No Select Agents)

Resource Sharing Plans:
Acceptable

Authentication of Key Biological and/or Chemical Resources:
Not Applicable (No Relevant Resources)

Budget and Period of Support:
Recommend as Requested:

CRITIQUE 3

Significance: 4
Investigator(s): 4
Innovation: 4
Approach: 4
Environment: 3

Overall Impact: This R34 application proposes to develop and conduct a preliminary randomized control trial of testing an eHealth enhanced, adapted navigator-led substance use and HIV-prevention

referral and linkage intervention (i.e., PrEP and substance use treatment services) for young adults involved in the criminal justice system (CJI-young adults supervised in the community) in San Francisco. Some strengths of the application include the solid preliminary data by the investigator's ongoing group along with the other co-investigators' previous work with a similar target population, having a strong collaboration with the co-investigators and an established academic-community partnership with stakeholders, and well thought out procedures for implementation components, all of which indicate the feasibility of the proposed project. Some potential weaknesses would be the limited scope from simply adapting an existing evidence-based navigator model from the ongoing project without a conceptual justification, the incorporation of eHealth and digital technology as implementation strategies, and the incremental scientific merit to implementation science. Additional weaknesses would be the lack of a sound conceptual framework in regards to examining the multiple factors that relate to the study outcomes, and an analytic approach that seems to essentially be to take a "kitchen-sink" type of approach and see what pops up as "significant". Such an exploratory analytical approach would generate some large challenges in translating the study outputs for incorporation into intervention components specific to this study population and to be combined into an eHealth intervention strategy. Finally, another weakness would be the lack of Innovation in implementation components or in strategies that are specific to this proposed project. These limitations were the main drivers of my overall score.

1. Significance:

Strengths

- The application is informed by strong preliminary data from the PI's ongoing project and other projects conducted by the investigative team.
- The application addresses an important problem by proposing eHealth-enhanced patient navigation for criminal-justice involved youth. The proposed intervention study would advance the field of implementation science and demonstrate the importance of community-based approach to foster effective linkage to substance use treatment and HIV-prevention service for this population.

Weaknesses

- There is a concern about the limited enhancement of scientific knowledge and technical capabilities of the preventive intervention components. It is not clear what, if any, would qualify as a unique contribution of this proposed adaptation intervention study to eHealth and digital health and health disparity.

2. Investigator(s):

Strengths

- The PI (Dr. Dauria) is a junior researcher with behavioral science background in the reach areas of Criminal justice, substance abuse and HIV, and currently serves as PI on NIDA funded R34 project of relevant topic with other Co-I also listed on this project.
- The project team is composed of a multidisciplinary team with expertise in psychology, behavioral intervention, criminal justice involved populations, substance use, implementation science with mHealth experience in the field of health disparity and HIV presentation and care continuum.

Weaknesses

- Project team is heavily weighted towards expertise in psychological and behavioral intervention backgrounds but is lacking in clinical expertise and eHealth. The investigative team would be benefited by the addition of more complementary expertise to collectively contribute their knowledge and skills in addressing HIV prevention among CJI youths.

3. Innovation:

Strengths

- The intervention component of peer-led navigation combined with eHealth approach for HIV prevention to support behavioral health care management targeting CJI youth population would be somewhat innovative.

Weaknesses

- Although the proposed integration of eHealth into existing intervention component would be innovative, it is not clear how the proposed adapted eHealth intervention from technological components, could increase digital communication and increase effectiveness of supporting behavioral health care engagement in the settings of CJI youth population.

4. Approach:

Strengths

- The application is informed by ongoing work led by the PI and other relevant intervention studies by the investigative team related to eHealth behavioral intervention with similar study population.
- The intervention approach is guided by the Consolidated Framework for Implementation Research, and the research strategies are well thought out for delineating the procedures.
- The applicants have forged strong partnerships with the academic-community and with the San Francisco Adult probation department, which ensure the feasibility of the proposed study.

Weaknesses

- The application does not describe a conceptual framework for patient navigation method.
- Related to the above issue, the scope of work may be narrow, as the application is heavily based on the existing and ongoing intervention studies for similar population. An intervention adaptation would greatly strengthen the implementation science research on HIV/Substance use intervention via the integrated eHealth virtual service delivery methods.
- The research strategy regarding data analysis lacks a sound conceptual grounding that guides the analysis of associations between/among multiple factors and study outcomes with potential mediator effects. The proposed analytical approach would pose some challenges in translating the study outputs into intervention components and eHealth intervention strategies.

5. Environment:

Strengths

- UCSF provides excellent institutional support, equipment and other resources, especially with the presence of Digital Health Core and IT, Data management systems. This should assist the investigative team in achieving the proposed aims.

Weaknesses

- None noted.

Study Timeline:

Strengths

- The proposed timelines of activities are reasonable and justified. These timelines show sufficient time for start-up activities, reasonable anticipated enrollment rates given the estimated eligible pool (508 individuals), well thought-out planned follow-up assessment, adapting existing resources, refining and testing it, assessing the feasibility and acceptability, and analyzing the collected data.

Weaknesses

- None noted.

Protections for Human Subjects:

Acceptable Risks and/or Adequate Protections

- The proposed study conducts focus groups and pilot efficacy trial for the criminal-justice involved young adults (ages of 18-29) on SF ADP probation. The study also conducts individual interviews with staff at SF ADP and PrEP navigators.

Data and Safety Monitoring Plan (Applicable for Clinical Trials Only):

Acceptable

Inclusion Plans:

- Sex/Gender: Distribution justified scientifically
- Race/Ethnicity: Distribution justified scientifically
- For NIH-Defined Phase III trials, Plans for valid design and analysis: Not applicable
- Inclusion/Exclusion Based on Age: Distribution justified scientifically
- The recruitment does not include minors.

Vertebrate Animals:

Not Applicable (No Vertebrate Animals)

Biohazards:

Not Applicable (No Biohazards)

Applications from Foreign Organizations:

Not Applicable (No Foreign Organizations)

Select Agents:

Not Applicable (No Select Agents)

Resource Sharing Plans:

Acceptable

- Guide and coding structure of qualitative data from interview and focus group will become publicly available, in addition to the other materials related to the navigator manual.

Authentication of Key Biological and/or Chemical Resources:
Not Applicable (No Relevant Resources)

Budget and Period of Support:
Recommend as Requested:

THE FOLLOWING SECTIONS WERE PREPARED BY THE SCIENTIFIC REVIEW OFFICER TO SUMMARIZE THE OUTCOME OF DISCUSSIONS OF THE REVIEW COMMITTEE, OR REVIEWERS' WRITTEN CRITIQUES, ON THE FOLLOWING ISSUES:

PROTECTION OF HUMAN SUBJECTS: ACCEPTABLE

INCLUSION OF WOMEN PLAN: ACCEPTABLE

INCLUSION OF MINORITIES PLAN: ACCEPTABLE

INCLUSION ACROSS THE LIFESPAN: ACCEPTABLE

COMMITTEE BUDGET RECOMMENDATIONS: The budget was recommended as requested.

Footnotes for 1 R34 DA054853-01; PI Name: Dauria, Emily F

NIH has modified its policy regarding the receipt of resubmissions (amended applications).See Guide Notice NOT-OD-18-197 at https://grants.nih.gov/grants/guide/notice-files/NOT-OD-18-197.html. The impact/priority score is calculated after discussion of an application by averaging the overall scores (1-9) given by all voting reviewers on the committee and multiplying by 10. The criterion scores are submitted prior to the meeting by the individual reviewers assigned to an application, and are not discussed specifically at the review meeting or calculated into the overall impact score. Some applications also receive a percentile ranking. For details on the review process, see http://grants.nih.gov/grants/peer_review_process.htm#scoring.
